# Cultivation has selected for a wider niche and large range shifts in maize

**DOI:** 10.7717/peerj.14019

**Published:** 2022-09-22

**Authors:** Rujing Yang, Runyao Cao, Xiang Gong, Jianmeng Feng

**Affiliations:** Department of Life Science and Agronomy, Dali University, Dali, Yunnan, China

**Keywords:** Human cultivation, Maize, Niche shifts, Range shifts, Wild progenitors

## Abstract

**Background:**

Maize (*Zea mays* L.) is a staple crop cultivated on a global scale. However, its ability to feed the rapidly growing human population may be impaired by climate change, especially if it has low climatic niche and range lability. One important question requiring clarification is therefore whether maize shows high niche and range lability.

**Methods:**

We used the COUE scheme (a unified terminology representing niche centroid shift, overlap, unfilling and expansion) and species distribution models to study the niche and range changes between maize and its wild progenitors using occurrence records of maize, lowland teosinte (*Zea mays* ssp. *parviglumis*) and highland teosinte (*Zea mays* ssp. *mexicana*), respectively, as well as explore the mechanisms underlying the niche and range changes.

**Results:**

In contrast to maize in Mexico, maize did not conserve its niche inherited from lowland and highland teosinte at the global scale. The niche breadth of maize at the global scale was wider than that of its wild progenitors (ca. 5.21 and 3.53 times wider compared with lowland and highland teosinte, respectively). Compared with its wild progenitors, maize at global scale can survive in regions with colder, wetter climatic conditions, as well as with wider ranges of climatic variables (ca. 4.51 and 2.40 times wider compared with lowland and highland teosinte, respectively). The niche changes of maize were largely driven by human introduction and cultivation, which have exposed maize to climatic conditions different from those experienced by its wild progenitors. Small changes in niche breadth had large effects on the magnitude of range shifts; changes in niche breadth thus merit increased attention.

**Discussion:**

Our results demonstrate that maize shows wide climatic niche and range lability, and this substantially expanded its realized niche and potential range. Our findings also suggest that niche and range shifts probably triggered by natural and artificial selection in cultivation may enable maize to become a global staple crop to feed the growing population and adapting to changing climatic conditions. Future analyses are needed to determine the limits of the novel conditions that maize can tolerate, especially relative to projected climate change.

## Introduction

Maize (*Zea mays* L.) is one of the world’s four staple crops. It is one of the most widely distributed crops and is widely used as a food source for humans and animals, as well as a raw material for industrial products. It is the third most economically important crop after wheat and rice, with an average cultivation area of 1.29 × 10^8^ ha (estimates from 1970 to 2000) (Leff, Ramankutty & Foley 2004; *Food and Agriculture Organization, http://faostat.fao.org, FAO, Rome, Italy*). Around 9,000 years ago, maize was selected from its wild form to its cultivated form (Aguirre-Liguori, Aguirre-Planter & Eguiarte, 2016; Lombardo et al., 2020; Moreno-Letelier et al., 2020). It has since spread throughout the world through trade and colonization (Lombardo et al., 2020), and it has played an essential role in feeding a rapidly growing human population in the past. However, its ability to do so may be impaired by climate changes, especially if it has low climatic niche lability. In other words, low climatic niche lability could be closely associated with weak adaptation to climate changes and small range expansions, limiting the role of maize in feeding the global population. The niche shifts induced by maize cultivation therefore need significant attention.

Maize and its wild progenitors (lowland teosinte (*Zea mays* ssp. *parviglumis*) and highland teosinte (*Zea mays* ssp. *mexicana*)) share the same evolutionary origins (Doebley & Stec, 1993; Doebley, 2004), and hence probably share the same initial niche spaces. We can reasonably infer that the realized niches of maize may have been shaped by human activities (*e.g*., introduction, breeding, and cultivation), whereas the realized niche of the wild progenitors has been continually shaped by natural selection pressures. Therefore, maize and its wild progenitor provide an excellent opportunity to assess the effects of human cultivation on niche shifts. This topic has received much attention from researchers in recent decades. Previous studies have mainly focused on the effects of climate change on the species distributions (*e.g*., Hufford et al., 2012; Ureta et al., 2012; Hellin, Bellon & Hearne, 2014; Aguirre-Liguori et al., 2019) and associated with ancestral introgression in maize and its wild progenitors (*e.g*., Aguirre-Liguori, Aguirre-Planter & Eguiarte, 2016; Le Corre et al., 2020; Calfee et al., 2021). For example, Aguirre-Liguori et al. (2019) predicted that climate change will disrupt patterns of local adaptation in wild and cultivated maize. Additionally, Le Corre et al. (2020) found that adaptive crop-to-wild introgression has induced both rapid adaptation to a new climatic niche and acquisition of herbicide resistance. However, global climatic niche shifts between maize and its wild progenitor require increased attention.

Humans are altering the biological world at an increasingly rapid rate (Pavlik et al., 2021). Indeed, anthropogenically mediated changes in terrestrial ecosystems during the Late Holocene have been even more rapid than changes observed during the end of the last glacial period (Mottl et al., 2021). Human activities not only affect biodiversity but also cause changes in species distributions (Xu et al., 2019).

To increase crop yields and meet the growing demand for food, crop species have often been introduced to new areas, and this has resulted in large changes in the distributions of crops (van Kleunen et al., 2015) and the adaptability of crop species to novel climatic conditions, which has likely led to the expansions of their realized niches.

In the coming decades, agriculture will need to face the challenge of meeting growing demands for food, fiber, and animal feed against the background of anthropogenically driven climate change (Bailey-Serres et al., 2019). One major issue requiring attention is how cultivation affects the adaptability of crop species to global environmental changes; whether the end is near for single staple crops such as wheat, maize, rice, and soybeans remains to be seen. There is therefore an urgent need to clarify the impacts of cultivation on the adaptability of crop species to global environmental changes (Koch et al., 2022).

The ecological niche is a key concept in ecology and biogeography (Wiens, 2011). While the niche can be interpreted or examined in a variety of ways, the realized niche, that is a part of the fundamental niche actually occupied by the species delimited by constraints of biotic and abiotic interactions, as well as dispersal ability (Wiens & Graham, 2005), provides an essential frame for understanding species distributions and the adaptation of species to the environment. The realized niche can be approximated by investigating species–environment associations across time and space (Broennimann et al., 2012; Guisan et al., 2014).

One of the important topics on the realized niche is whether a species conserves its initial niche space in novel environmental conditions or not. Niche conservatism is a central assumption in species distribution models (Pearman et al., 2008; Warren, Glor & Turelli, 2008). However, controversy remains regarding whether species niches are conserved across time and space (niche conservatism hypothesis). The relative roles of biological invasions and crop cultivation in driving niche shifts have not yet been evaluated. Most studies of invasive species have indicated that the climatic niche is conserved (Petitpierre et al., 2012; Strubbe et al., 2013; Beukema et al., 2018), but other studies have failed to find support for climate niche conservatism (Goncalves et al., 2014; Hernandez-Lambrano, Gonzalez-Moreno & Sanchez-Agudo, 2017; Datta, Schweiger & Kuhn, 2019). This uncertainty greatly reduces our confidence in using species distribution models (SDMs) to predict species distributions under scenarios of global change.

One explanation for the contrasting conclusions may be that different techniques have been used to verify the niche conservatism hypothesis and evaluate species niche dynamics (Liu et al., 2020). Although there is currently no consensus on which technique is optimal in most contexts, the COUE scheme, which uses niche expansions (E), niche unfilling (U), and niche stability (S), as well as the niche similarity index and breadth ratio to measure niche shifts, is currently the gold standard to address niche conservatism (Guisan et al., 2014; Atwater, Ervine & Barney, 2018), indeed, it has become the gold standard for analyzing niche dynamics, possibly because it possesses the simplicity and effectiveness of the ordination approach and also accounts for the biases caused by sampling effort and spatial resolution (Petitpierre et al., 2012; Guisan et al., 2014; Liu et al., 2020). In addition, the COUE scheme can be used to determine whether the niche of a species is conserved, as well as to measure shifts in niche breadth and position (Wiens et al., 2010; Guisan et al., 2014; Liu et al., 2020). However, most studies of niche dynamics using the COUE scheme have been conducted on plant invasions; by contrast, few studies have been conducted on cultivated crops, although Yang et al. (2021) found that the niche shifts of wheat were closely associated with human cultivation.

Brown (1984) suggested that when a species can exploit a wider variety of resources and maintain its viable populations under a wider range of conditions, niche and range expansion could be observed. This leads to the prediction that the niche space of a species should be positively correlated with species range size. For example, contractions in range size might be accompanied by decreases in niche breadth and *vice versa* (Morin & Lechowicz, 2013; Slatyer, Hirst & Sexton, 2013; Sheth, Morueta-Holme & Angert, 2020; Bernard et al., 2021). The range dynamics of invasive species have attracted increased research attention in recent years. For example, Osland & Feher (2020) observed that winter warming may promote the range expansions of tropical invasive species. Bellard et al. (2013) found that climate changes might cause range expansions in temperate species at higher latitudes but range contractions in tropical species. However, most projections of species range shifts under climate change scenarios have assumed that the niches of species are conserved. We assumed that the niches of species have been and continue to be modified by human activities (*e.g*., species introduction, cultivation, and breeding), and these niche shifts can have substantial effects on species range dynamics. However, few studies have examined range dynamics under scenarios of niche shifts in cultivated crop species.

Cultivation is one of the most pervasive human activities on the planet. The introduction, breeding, and planting of species enable them to occur in areas outside their native ranges (Milla, 2020) and provide opportunities for niche shifts, which can lead to range shifts. Although various species have been used to test whether ancestral niches are conserved, most studies have focused on invasive species (Tingley et al., 2014; Atwater, Ervine & Barney, 2018; Liu et al., 2020); few studies to date have evaluated climatic niche conservatism in crop species at a global scale. Crop species and their wild progenitors have the same evolutionary origins and highly similar genes. The only difference is that the niches of wild progenitors have been subjected to natural selection pressures; by contrast, the niches of crop species have been shaped by artificial selection, in addition to natural selection. The new ecological pressures faced by cultivated plants can be, among others, water availability modified by irrigation, reduction in natural selective pressures associated with diseases, pests, and other herbivores, and even complete changes in the natural enemies in new environments.This provides an excellent opportunity to investigate the effects of crop cultivation on niche shifts.

Here, we used the COUE scheme and SDMs to study the niche and range changes between maize and its wild progenitors using global occurrence records of maize and its wild progenitors, as well as those in Mexico. Specifically, we aimed to explore the niche and range changes between maize and its wild progenitors and to identify the factors that controlled niche changes. The results of this study provide new insight into the effects of human cultivation on species niche and range changes, as well as the underlying mechanisms responsible for the emergence of maize as a global staple crop. Our study also provides essential information that could help managers and policymakers develop strategies to enhance maize production against the background of global climate change.

## Materials and Methods

### Species occurrence data

We standardized species names of maize and its wild progenitors following the Taxonomic Name Resolution Service (TRNS, http://tnrs.iplantcollaborative.org/). Occurrence records of maize and its wild progenitors (*i.e*., lowland teosinte (*Zea mays* ssp. *parviglumis*) and highland teosinte (*Zea mays* ssp. *mexicana*)) were retrieved mainly from the GBIF (Global Biodiversity Information Facility, Copenhagen, Denmark, https://www.gbif.org/), GRIN (Germplasm Resource Information System, Beltsville, Maryland, http://www.grin-global.org), GCDT (Global Crop Diversity Trust, Bonn, Germany, http://www.croptrust.org), FAO (http://www.FAO.org), MaizeGDB (Maize genetics and genomics database, http://www.maizegdb.org), and the CHV (Chinese Virtual Herbarium, https://www.cvh.ac.cn/) accessed from May 1^st^, 2020 to May 6^th^, 2022. In addition to records from online sources, we also conducted an extensive literature search for occurrence records of these two species in Web of Science and Google Scholar using topic search strategy.

**Figure 1:**
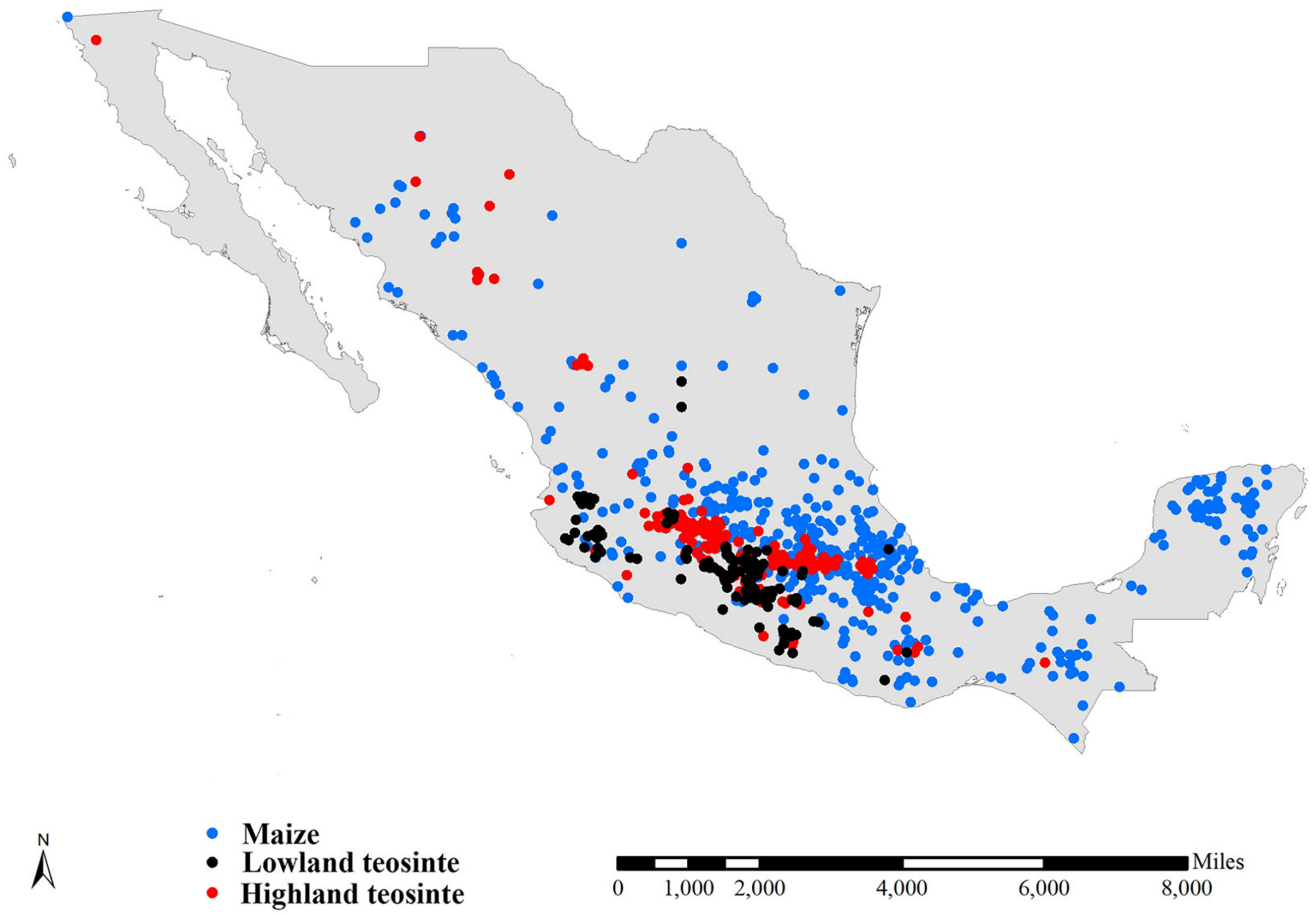
Occurrence records of maize, lowland teosinte, and highland teosinte in Mexico. We spatially rarefied occurrence records with a radius of 10 km to reduce spatial autocorrelation, and we obtained 449, 122, and 168 occurrence records maize, lowland teosinte, and highland teosinte in Mexico, respectively. Blue, black and red points indicate occurrence records of maize, lowland teosinte, and highland teosinte, respectively.

**Figure 2:**
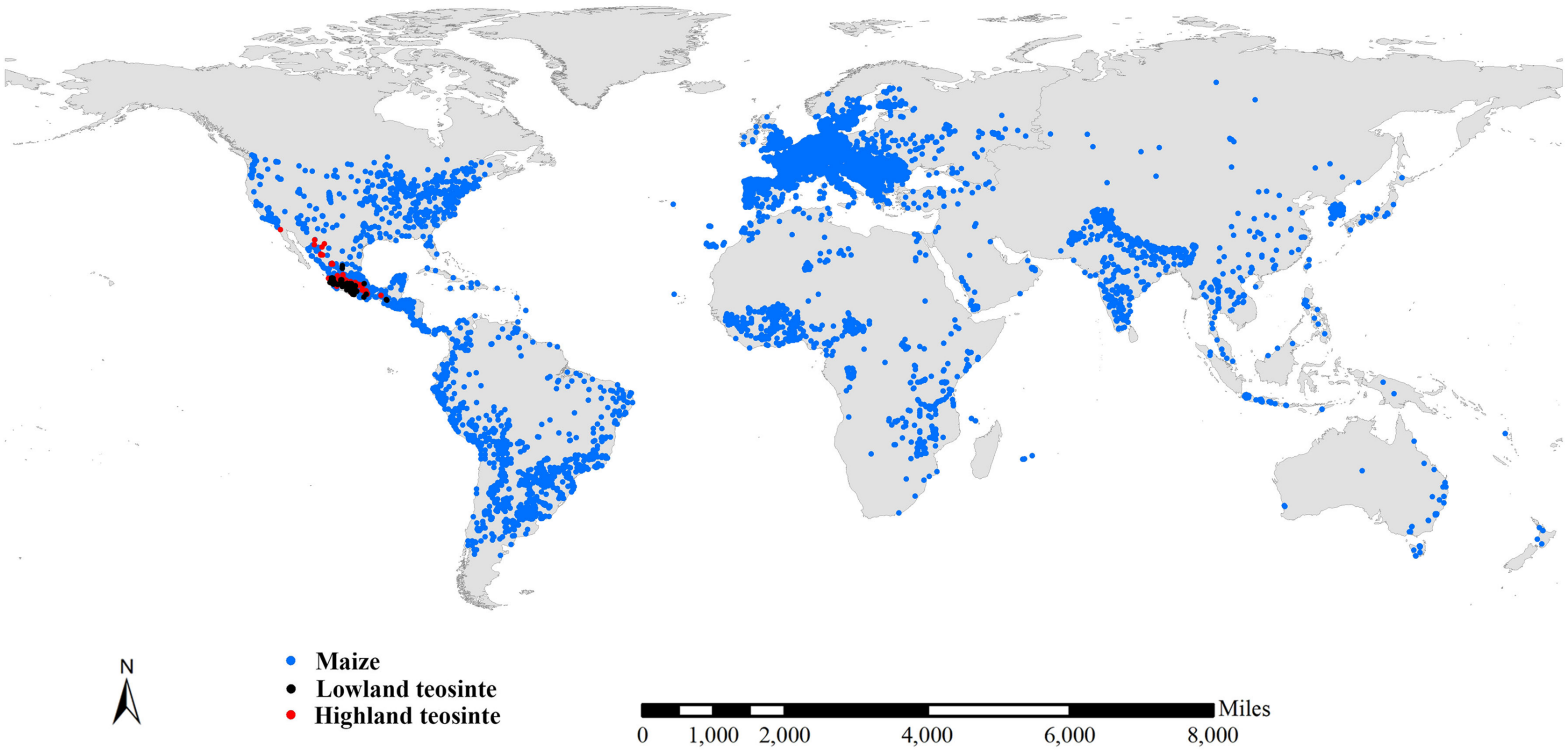
Global occurrence records of maize, lowland teosinte, and highland teosinte. We spatially rarefied occurrence records with a radius of 10 km to reduce spatial autocorrelation, and we obtained 7,291, 124, and 185 occurrence records of maize, lowland teosinte, and highland teosinte, respectively. Blue, black and red points represent occurrence records of maize, lowland teosinte, and highland teosinte, respectively.

We compiled three preliminary global datasets consisting of 15,723, 743 and 751 distinct occurrence records for maize, lowland teosinte and highland teosinte, respectively. We also compiled three preliminary global datasets consisting of 793, 732 and 726 distinct occurrence records for maize, lowland and highland teosinte in Mexico, respectively. To refine the datasets, we deleted records for which the latitude and longitude uncertainty was greater than 10 km and removed duplicate latitude and longitude entries. As sampling bias has an adverse effect on the results of SDMs (Peterson, 2011), we used SDMtoolbox v2.4 (Brown, 2014; Brown, Bennett & French, 2017) to spatially rarefy occurrence records with a radius of 10 km to account for spatial autocorrelation. After spatial rarefaction, we were left with 449, 122 and 168 occurrence records in Mexico, respectively (Fig. 1). At global scale, we obtained 7,291 records of maize, 124 records of lowland teosinte and 185 records of highland teosinte (Fig. 2).

### Climatic predictors

We downloaded 19 bioclimatic factors from WorldClim 2.1 (https://www.worldclim.org/data/worldclim21.html) (Fick & Hijmans, 2017) at a spatial resolution of 5 arc-minutes (ca. 10 km) for 1970–2000, which included 11 and 8 water and thermal-related variables, respectively. Additionally, we estimated GDD (with 5 °C as the base temperature) based on 12 monthly mean temperatures with the same spatial resolution from WorldClim 2.1 (https://www.worldclim.org/data/worldclim21.html) (Fick & Hijmans, 2017) using a method proposed by Svenning & Skov (2004). The aridity index (Arid) at the same spatial resolution was calculated as the ratio of annual precipitation to annual potential evapotranspiration (Budyko, 1974).

### Analysis of niche dynamics

Using global occurrence records of maize and its wild progenitors, we investigated the niche shifts between them at the global scale. We used the COUE scheme (Broennimann et al., 2012) to investigate the niche changes between maize and each of its wild progenitors using the “ecospat” package in R (3.6.3) (Di Cola et al., 2017). In the COUE scheme (Warren, Glor & Turelli, 2008; Petitpierre et al., 2012; Guisan et al., 2014), we first conducted principal component analysis (PCA) on the 21 climatic predictors to construct a two-dimensional environmental space represented by a 100 × 100 grid. We then used the kernel density function to estimate the record density of maize and its wild progenitors in each cell.

We divided the environmental space occupied by maize and each of its wild progenitors into Stability (S), Unfilling (U), and Expansion (E). Stability denoted niche spaces occupied by maize and each of its wild progenitors. Unfilling indicated niche spaces occupied only by each of the wild progenitors but not by maize. Expansion indicated an ecological niche occupied exclusively by maize. The breadth of the native niche (*BN*, occupied by each of the wild progenitors) and the breadth of the introduced niche (*BI*, occupied by maize) were expressed as: $BN = U + S$ and $BI = E + S$, respectively. The breadth ratio (*BR*) between each of the wild progenitors and maize was expressed as follows:

$BR=\frac{BI}{BN}.$

When the ecological niches of the wild progenitors and maize have the same breadth, *BR* = 1, and when *BR* > 1, the niche breadth of the wild progenitors is narrower than that of maize.

Sørensen’s similarity index (*SI*, Baselga, 2017; Liu et al., 2020) was used to determine changes in the niche positions between each of the wild progenitors and maize:

$SI=\frac{2S}{BN+BI}.$

*SI* ranges from 0 to 1; when *SI* > 0.5, the wild progenitors and maize occupy similar niche positions, whereas when *SI* < 0.5, the wild progenitors and maize occupy different niche positions. In sum, when *BR* > 1 and *SI* < 0.5, maize does not conserve the niche of the wild progenitors, and the niche conservatism hypothesis is rejected (Liu et al., 2020).

Using occurrence records of maize at global scale and those of the wild progenitors, the COUE scheme was used to determine the niche changes between maize and the wild progenitors at a global scale. Using the COUE scheme and the occurrence records in Mexico, we also investigated the niche changes between maize and each of the wild progenitors in Mexico.

We identified the most important predictor that contributed to principal component 1 (PC1) and to principal component 2 (PC2) of the PCA, extracted their values in the climatic predictor layers based on the locations of the occurrence records, and then used independent-samples *t*-tests to compare the mean values between maize and its wild progenitors. We also extracted the maximum and minimum values of 21 climatic factors for the maize occurrence records at the global scale and for its wild progenitors, and we used paired-samples *t*-tests to compare the ranges of the 21 climatic factors between maize and its wild progenitors. In these analyses, we used the feature scaling method proposed by Becker, Chambers & Wilks (1988) to standardize our datasets.

### Projecting potential species ranges

We used global occurrence records of maize and its wild progenitors to project their global potential ranges. In addition, we also used the occurrence records in Mexico to project their potential ranges in Mexico. We used biomod v.2.0 (Thuiller et al., 2009), a platform for generating SDMs, to predict the potential species ranges. To account for potential spurious effects of different techniques for generating SDMs, we used seven different algorithms in the biomod v.2.0 platform (Thuiller et al., 2009) and retained only those SDMs with true skill statistics (TSS) greater than 0.6 or areas under the receiver operating characteristic curve (AUC) greater than 0.8 (*e.g*., Gallien et al., 2012). The seven ENM algorithms used included artificial neural network (ANN), maximum entropy model (MaxEnt), random forest (RF), generalized additive model (GAM), flexible discriminant analysis (FDA), classification tree analysis (CTA), and generalized boosting model (GBM) (Thuiller et al., 2009). In addition, we used an ensemble approach wherein a weight (proportional to the TSS) was assigned to each algorithm’s projection to obtain the central tendency of the SDMs (Araujo & New, 2007). Per the requirements of SDMs, we generated two sets of pseudo-absences (PAs) by retrieving random points across the globe. According to Barbet-Massin et al. (2012), we randomly selected an equal number of PAs if the number of presence records was greater than 1,000 or 1,000 PAs if the number of presence records was less than 1,000. The threshold of the sensitivity-specificity sum maximization approach (MSS threshold) was used to determine the potential ranges of maize and its wild progenitors. We estimated the ratios of potential ranges of maize to those of its wild progenitors at the global scale and in Mexico, and we referred to these ratios as range ratios.

Hargrove, Hoffman & Hessburg (2006) proposed a quantitative goodness of fit (GOF) method that shows the degree of spatial concordance between two categorical maps with high robustness. In the present study, if the potential ranges between maize and its wild progenitors were projected in similar regions and sizes, showing high spatial overlap, GOF would show high values; otherwise, values would be low. We therefore used Mapcurve to compare the spatial patterns of the potential ranges between maize and its wild progenitors by calculating GOF as follows:

$GOF = \sum\limits_{i = 1}^n {\displaystyle{{{C_i}_{}} \over {{B_i} + {C_i}}}}\ *\ \displaystyle{{{C_i}_{}} \over {{A_i} + {C_i}}}$

where *A* and *B* are the numbers of grid cells of the potential ranges of maize and its wild progenitors, respectively, *C* is the intersection of the two ranges, and *n* is the number of categories in the maps.

## Results

**Figure 3:**
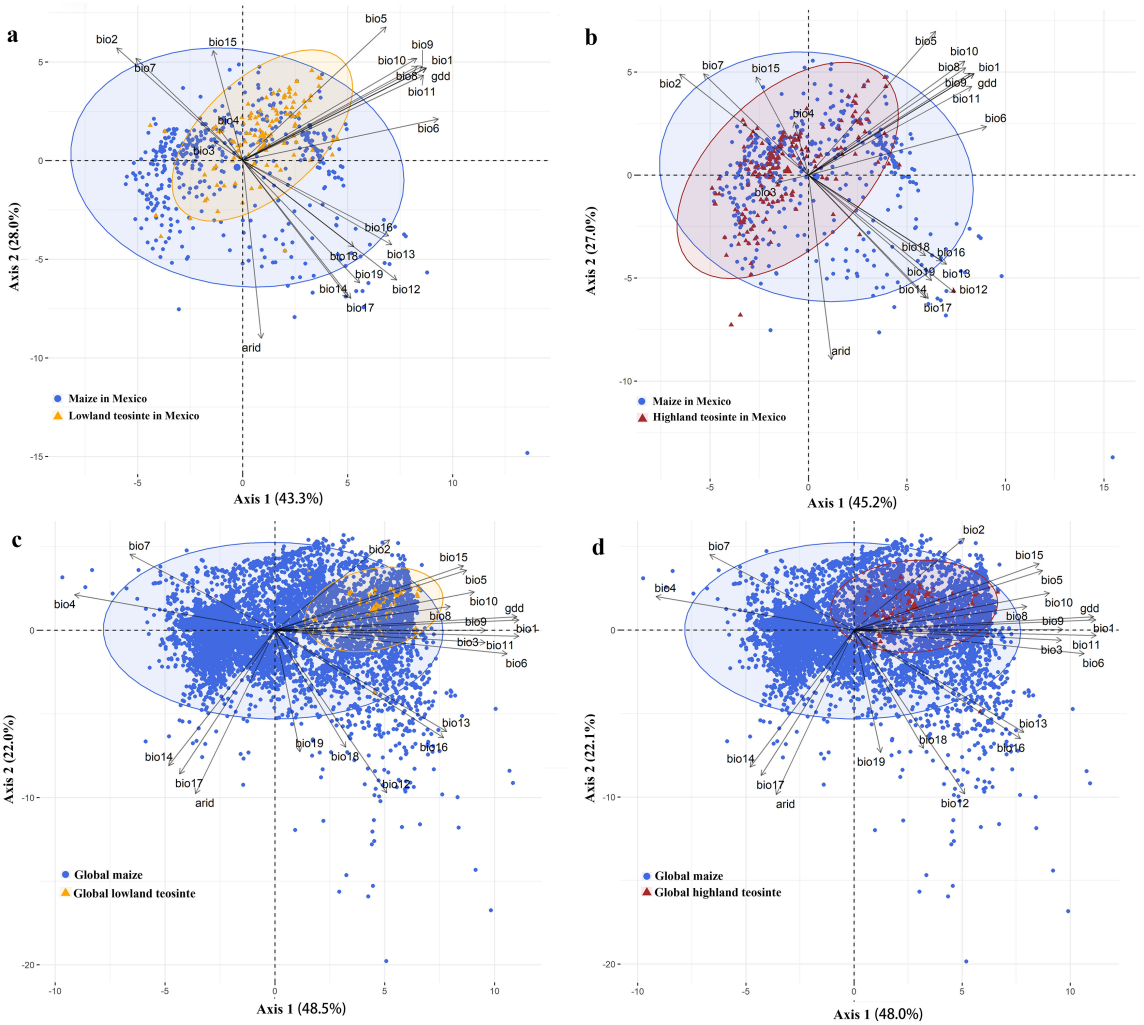
Principal component analysis of the climatic variables in two-dimensional niche space. (A–D) show the principal component analysis of the climatic variables between maize and lowland teosinte in Mexico, and maize and highland teosinte in Mexico, respectively, maize and lowland teosinte at the global scale, maize and highland teosinte at the global scale, respectively.

In Mexico, PC1 (43.3%) and PC2 (28.0%) of the PCA in the COUE scheme for maize and lowland teosinte explained most of variation among all climatic predictors (71.3%), and therefore the first two PC axes in the COUE scheme were responsible for most of niche space of lowland teosinte and maize (Fig. 3A). The major climatic predictors for PC1 were min temperature of the coldest month and growing degree days, and those for PC2 were arid index and precipitation of the driest quarter (Fig. 3A). Also in Mexico, PC1 (45.2%) and PC2 (27.0%) of the PCA were responsible for most variation among all climatic predictors (72.2%) for highland teosinte and maize, and the major climatic predictors for PC1 were min temperature of the coldest month and growing degree days, and those for PC2 were arid index and max temperature of the warmest month (Fig. 3B).

At the global scale, PC1 (48.5%) and PC2 (22.0%) of the PCA in the COUE scheme for the niche shifts between maize and lowland teosinte explained most of the variation among the 21 climatic predictors (70.5%), suggesting that the PC axes in the COUE scheme delimited most of the niche space of lowland teosinte and maize (Fig. 3C). PC1 was interpreted as a thermal axis because temperature-related variables, such as mean temperature of the coldest quarter, followed by mean annual temperature and growing degree days (Fig. 3C), loaded most heavily on PC1. PC2 was closely correlated to variables associated with water availability, including arid index, followed by annual precipitation and precipitation of the driest quarter (Fig. 3C). In sum, thermal and water variables were the main factors affecting the niche changes between maize and lowland teosinte, and thermal variables had a particularly strong effect.

At the global scale, PC1 (48.0%) and PC2 (22.1%) of the PCA in the COUE scheme for maize and highland teosinte explained most of the variation among the 21 climatic predictors (70.1%), suggesting that the PC axes in the COUE scheme delimited most of the niche space of highland teosinte and maize (Fig. 3D), and the major climatic predictors were same as those that explained the niche differences between maize and lowland teosinte.

**Figure 4:**
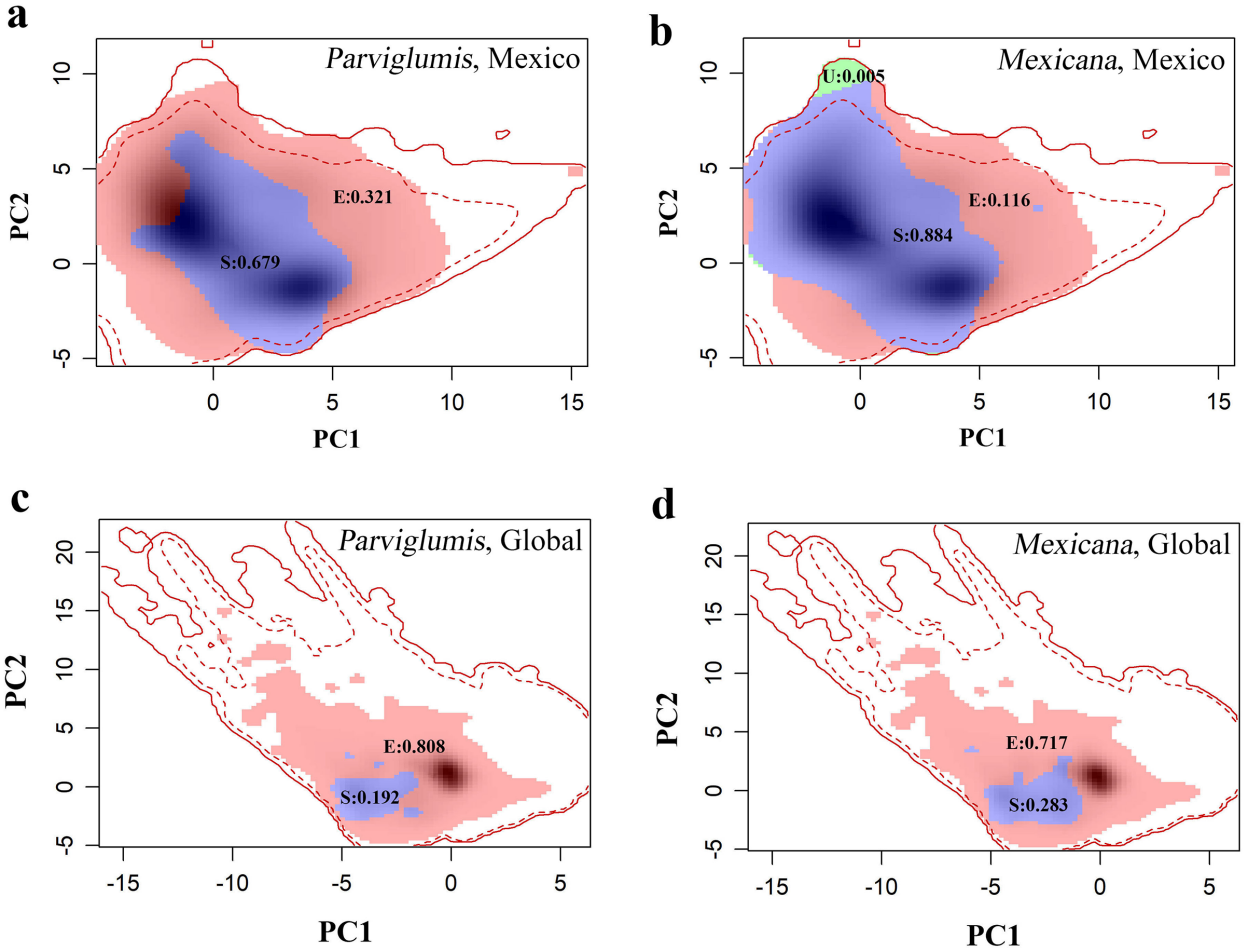
(A–D) COUE scheme analysis of niche dynamics between maize and its wild progenitors.

In Mexico, niche expansion, niche stability, and niche unfilling values in the niche dynamics between maize and lowland teosinte (and between maize and highland teosinte, in parenthesis) were 0.32 (0.12), 0.70 (0.88) and 0.00 (0.005), respectively (Figs. 4A and 4B). In Mexico, the breadth ratio and niche similarity were 1.47 (1.12) and 0.81 (0.94), respectively, suggesting that in general terms maize conserves the niche inherited from its wild progenitors. At the global scale, niche dynamics analyses showed that niche expansion, niche stability, and niche unfilling values between maize and lowland teosinte (highland teosinte) were ca. 0.81 (0.72), 0.19 (0.28), and 0.00 (0.00), respectively (Figs. 4C and 4D). In addition, the breadth ratio and niche similarity index were 5.21 (3.53) and 0.32 (0.44), respectively, suggesting that at a global scale, maize does not conserve the niche inherited from its wild progenitors.

**Figure 5:**
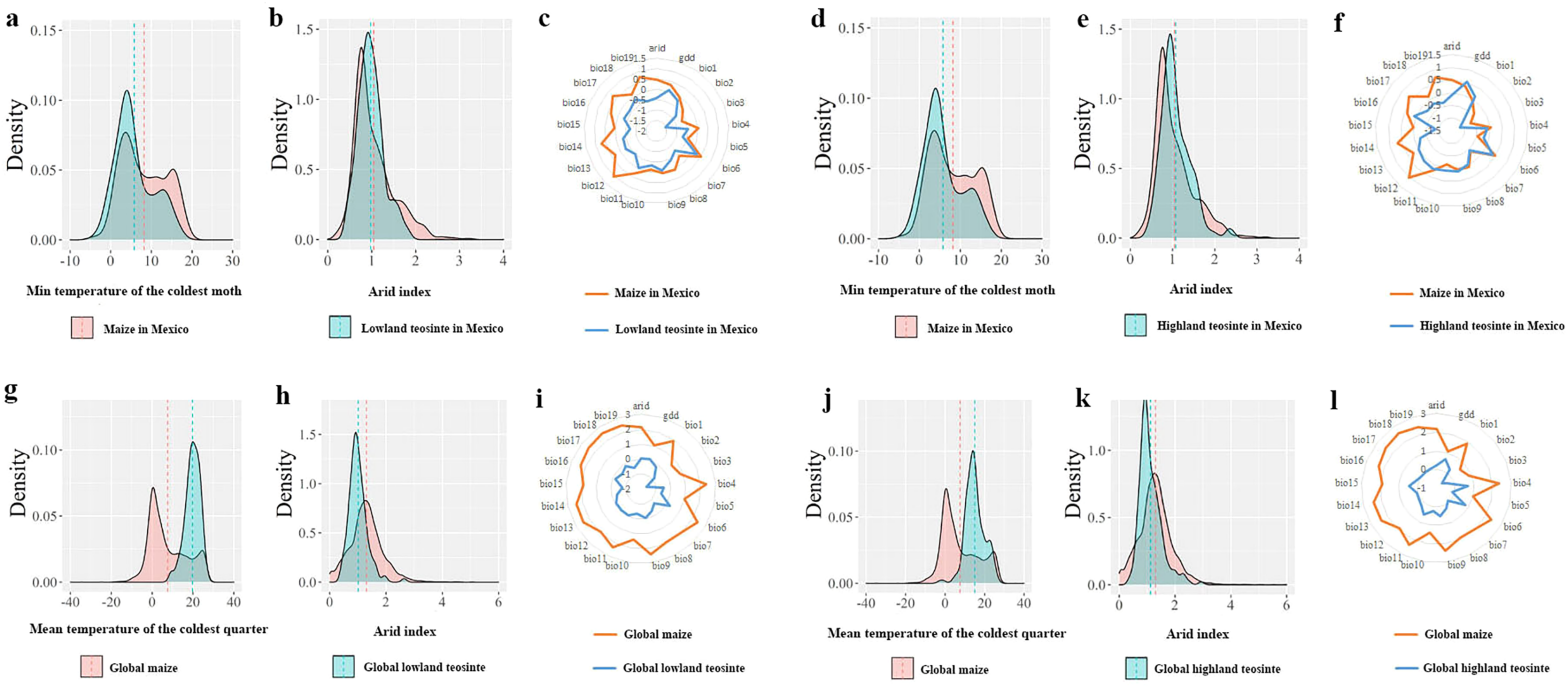
Comparisons of the major climatic predictors responsible for the niche shifts between maize and its wild progenitors. (A and D) show comparisons of the mean values of the min temperature of the coldest month between maize and lowland teosinte and between maize and highland teosinte in Mexico (*P* < 0.001), respectively. (B and E) show the comparisons of the arid index between maize and lowland teosinte (*P* = 0.052) and between maize and highland teosinte in Mexico (*P* = 0.35), respectively. (C and F) show the comparisons of the ranges of 21 climatic predictors between maize and lowland teosinte (*P* = 0.03) and between maize and highland teosinte in Mexico (*P* = 0.12), respectively. (G and J) show comparisons of the mean values of the mean temperature of the coldest quarter between maize and lowland teosinte and between maize and highland teosinte (*P* < 0.001) at the global scale, respectively. (H and K) show comparisons of the arid index between maize and lowland teosinte and between maize and highland teosinte (*P* < 0.001) at the global scale, respectively; (I and L) show comparisons of the ranges of 21 climatic predictors between maize and lowland teosinte (*P* = 0.003) and between maize and highland teosinte at the global scale (*P* = 0.004), respectively. In these analyses, we used the feature scaling method proposed by Becker, Chambers & Wilks (1988) to standardize our datasets.

In Mexico, independent-samples *t*-tests on the mean values of the min temperature of the coldest month between maize and its wild progenitors showed that maize tended to occur in regions with colder climates (maize *vs*. lowland teosinte: *P* < 0.001, Fig. 5A; maize *vs*. highland teosinte: *P* < 0.001, Fig. 5D), and there was no significant difference in the arid index between regions where maize and its wild progenitors were observed (maize *vs*. lowland teosinte: *P* = 0.052, Fig. 5B; maize *vs*. highland teosinte: *P* = 0.35, Fig. 5E). Additionally, paired-samples *t*-tests showed that the former tended to survive in regions with wider ranges of climatic predictors (ca. 5.71 and 1.92 times compared with lowland teosinte (*P* < 0.001, Fig. 5C) and highland teosinte (*P* < 0.001, Fig. 5F), respectively).

At a global scale, independent-samples *t*-tests showed that compared with its wild progenitors, maize tended to occur in regions with colder (maize *vs*. lowland teosinte: *P* < 0.001, Fig. 5G; maize *vs*. highland teosinte: *P* < 0.001, Fig. 5J) and wetter (maize *vs*. lowland teosinte: *P* < 0.001, Fig. 5H; maize *vs*. highland teosinte: *P* < 0.001, Fig. 5K) climates, and paired-samples *t*-tests showed that the ranges of 21 climatic predictors were significantly greater for maize than for its wild progenitors (ca. 4.51 and 2.40 times compared with lowland (*P* < 0.001, Fig. 5I) and highland teosinte (*P* < 0.001, Fig. 5L), respectively).

In Mexico, TSS and AUC in ensemble species distribution models for maize were 0.563 and 0.860, respectively, and those for lowland (highland) teosinte were ca. 0.896 (0.823) and 0.982 (0.968), respectively. At the global scale, the average TSS and AUC in the ensemble species distribution models for maize were ca. 0.725 and 0.939, respectively, and those for lowland (highland) teosinte in parenthesis were ca. 0.995 (0.976) and 0.999 (0.999), respectively. In sum, TSS and (or) AUC of the species distribution models exceeded their respective thresholds.

**Figure 6:**
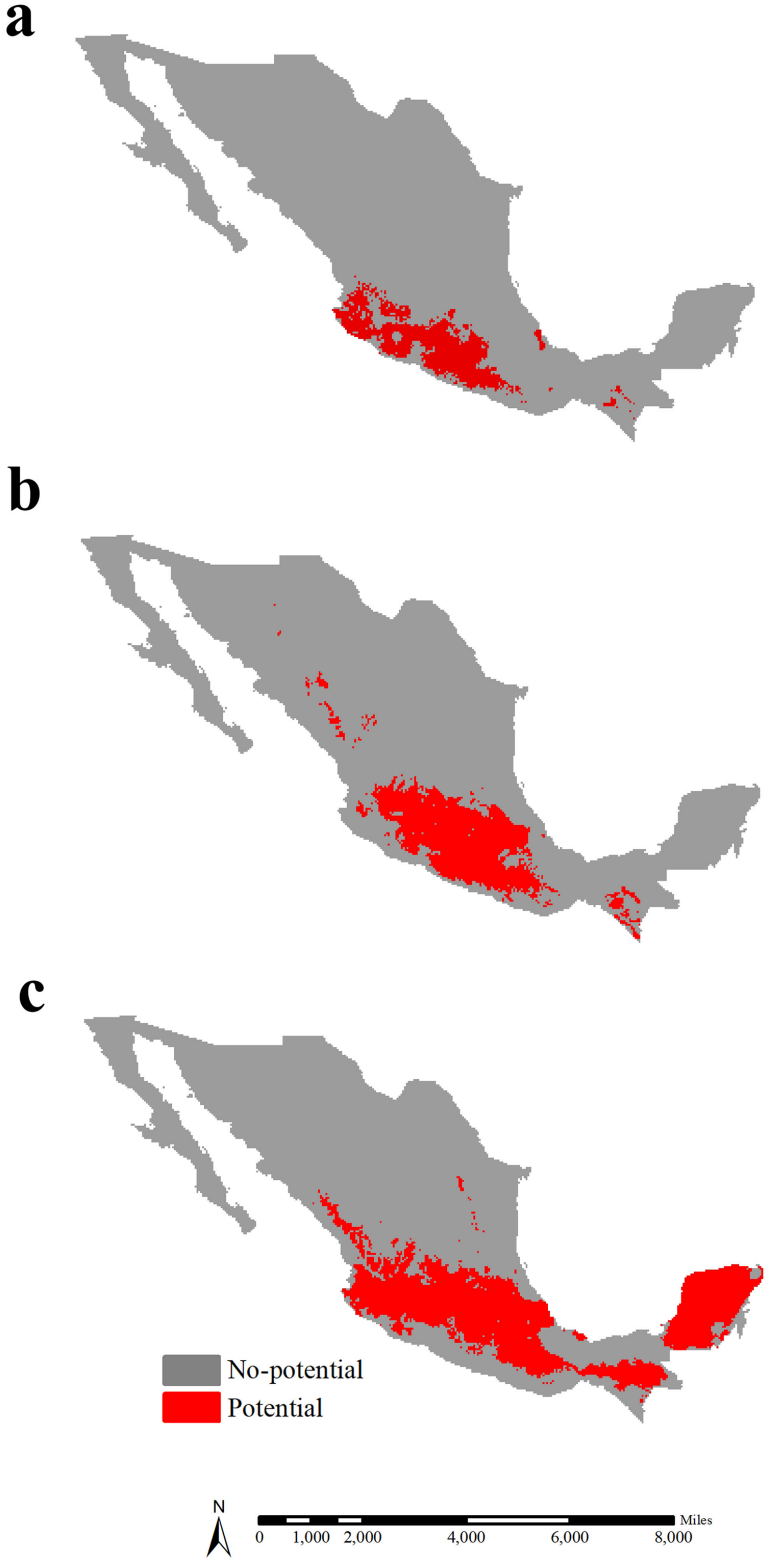
Potential ranges of maize and its wild progenitors in Mexico. (A-C) show the potential ranges for lowland teosinte, highland teosinte, and maize in Mexico, respectively. Red blocks indicate the potential ranges. The potential range of lowland teosinte was predicted to occur in southwestern Mexico. The potential range of highland teosinte was also observed in southwestern Mexico. The potential range of maize was mainly observed in southern Mexico.

In Mexico, the potential range of lowland teosinte was 113,751 km^2^, and was predicted to occur in southwestern Mexico (Fig. 6A). The potential range of highland teosinte was also observed in southwestern Mexico, covering 198,367 km^2^ (Fig. 6B). The potential range of maize covered 364,003 km^2^ and was mainly observed in southern Mexico (Fig. 6C). In Mexico, the GOF between the potential ranges of maize and lowland, highland teosinte was 0.071 and 0.124, respectively. The range ratios between maize and lowland, highland teosinte in Mexico were 3.20 and 1.83, respectively.

**Figure 7:**
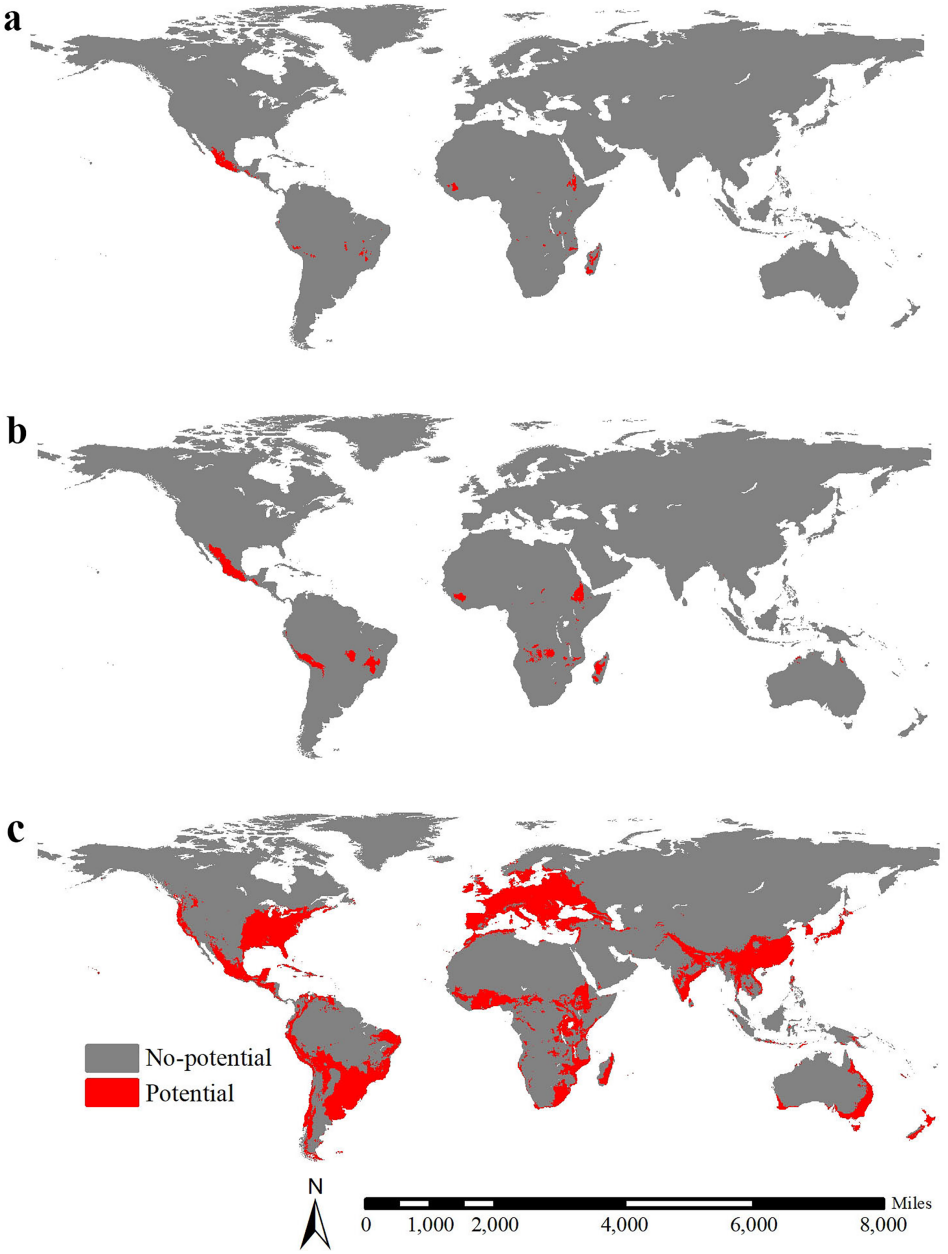
Global potential ranges of maize and its wild progenitors. (A-C) show the global potential ranges for lowland teosinte, highland teosinte, and maize, respectively. Red blocks indicate the potential ranges. The global potential range of lowland teosinte was mainly in Mexico, Madagascar, Guinea, and Ethiopia. The global potential range of highland teosinte was mainly in Mexico, Madagascar, Guinea, Ethiopia, Peru, Bolivia, and Brazil. At the global scale, the potential range of maize was mainly in Europe, the southern part of Australia, the Indochina Peninsula, South China, Japan, Korean Peninsula, the equatorial regions of Africa, eastern part of Africa, Madagascar, southern part of India, the coastal regions of South America, southern part of Brazil, Mexico, and the eastern coastal regions and south-eastern part of the United States.

The global potential range of lowland teosinte was 1,956,581 km^2^ (MSS threshold = 0.48), including areas mainly in Mexico, Madagascar, Guinea and Ethiopia (Fig. 7A). The global potential range of highland teosinte was 794,336 km^2^ (MSS threshold = 0.53), including areas mainly in Mexico, Madagascar, Guinea, Ethiopia, Peru, Bolivia and d Brazil (Fig. 7B). At the global scale, the potential range of maize was 26,298,202 km^2^ (MSS threshold = 0.47) and was mainly in Europe, the southern part of Australia, the Indochina Peninsula, South China, Japan, Korean Peninsula, the equatorial regions of Africa, eastern part of Africa, Madagascar, southern part of India, the coastal regions of South America, southern part of Brazil, Mexico, and the eastern coastal regions and south-eastern part of the United States (Fig. 7C). At a global scale, the GOF between the potential ranges of maize and lowland teosinte and between the potential ranges of maize and highland teosinte was 0.0056 and 0.028, respectively, suggesting strong range shifts. The range ratios between maize and lowland and between maize and, highland teosinte in Mexico were 13.44 and 33.11, respectively.

## Discussion

To our knowledge, this study represents the first global assessment of the influences of human cultivation on the niche and range dynamics between maize and its wild progenitors, influences that, to a great extent, have shaped its potential responses to climate change. Our results showed that the niche breadth of maize is wider than that of its wild progenitors at a global scale. After maize cultivation, the niches of lowland teosinte and highland teosinte were strongly modified by human cultivation, whereas teosintes continued to be exposed to natural selection pressures.

Humans first domesticated and cultivated maize approximately 9,000 years ago (Aguirre-Liguori, Aguirre-Planter & Eguiarte, 2016; Lombardo et al., 2020; Moreno-Letelier et al., 2020), and the divergence time of teosintes from their sister group occurred ca. 60,000–70,000 years ago (Ross-Ibarra, Tenaillon & Gaut, 2009*; Wang et al., 2017*). This indicates that crop cultivation has had a considerable effect on the (realized) niche expansion of maize from its wild progenitors, as the period of crop cultivation is only ca. 1/14 of the age of the teosinte lineage. This is consistent with the finding that niche shifts in introduced species can often occur much more rapidly than those in native species (Wiens et al., 2019) and suggests that the effect of crop cultivation on niche shifts can be substantial.

In addition, our results suggest that cultivation-induced niche shifts have enabled maize to be a global staple crop, feeding rapidly increasing populations and adapting to novel climatic conditions, which, to certain extent, was supported by Swarts et al. (2017) that artificial selection enable ancient maize’s adaptation to temperate North America. Our findings are also supported by a recent study showing that the niche conservatism hypothesis is not supported in wheat, and that the niche dynamics between wheat and its wild progenitors are closely associated with cultivation (Yang et al., 2021).

The wider niche breadth of maize relative to its wild progenitors may be closely related to the ability of crop cultivation to promote niche shifts compared with natural dispersal and selection. The introduction, breeding, and planting of maize have permitted it to occur in areas outside of its native range with novel climatic conditions. Maize was introduced to Europe after Europeans contacted the New World (Rebourg et al., 2003). Trade and colonization resulted in the introduction and migration of maize to various parts of the world (Mir et al., 2013). Breeding has since been performed using genomics and other molecular techniques to obtain new cultivars, including those with drought tolerance (Cooper et al., 2014) and insect resistance (Han, Jiang & Peng, 2016), a process that has probably contributed to the expansions of the niche space of maize. In addition to maize breeding, cultivation measures such as irrigation (Wang et al., 2021), fertilization, and other field management techniques (Jiang, Zhai & Zhang, 2019) can drive the niche expansion of maize.

The above observations are consistent with our findings that maize can survive in regions with wider ranges of climatic conditions than its wild progenitors, substantially expanding its realized niche. However, caution is needed in interpreting our observations because cultivated plants are very different from wild plants in their ecology, as they differ in their sensitivity to soil fertilization and degree of protection against natural enemies.

Our results show that maize does not conserve the niche space inherited from its wild progenitors, in contrast to the findings in invasive plant species by Liu et al. (2020) where only 0.2% of them fail to show niche conservatism. One possible explanation for this difference is the contrast in the time over which niche shifts have been able to occur between cultivated maize and invasive plants. The history of maize cultivation is much longer than the history of most plant invasions (ca. 9,000 *vs*. 500 years) (Aguirre-Liguori, Aguirre-Planter & Eguiarte, 2016; Liu et al., 2020; Lombardo et al., 2020; Moreno-Letelier et al., 2020), and this provides more time and opportunity for niche shifts to occur. In addition, maize may have had more opportunities for introduction and cultivation in regions beyond its original range compared with other invasive plant species. For example, maize cultivation measures such as irrigation (Wang et al., 2021), fertilization, and other field management techniques (Jiang, Zhai & Zhang, 2019) that should be important for the observed niche expansion of maize, whereas for most invasive plant species, the probability of obtaining these opportunities might be very low. These may explain the greater niche shifts in maize compared with other invasive plants. The lack of niche conservatism in maize may have contributed to its ability to become a staple crop since it was first cultivated approximately 9,000 years ago (Aguirre-Liguori, Aguirre-Planter & Eguiarte, 2016; Moreno-Letelier et al., 2020).

Niche stability and the niche similarity index at the global scale were lower compared with those in Mexico, whereas the breadth ratio exhibited the opposite pattern, suggesting stronger niche shifts at the global scale than those in Mexico. This contrast may be partially due to scale effects. At the global scale, maize could be introduced and cultivated in regions with diverse climatic conditions, facilitating its adaptation to novel climatic conditions different from those in its area of origin, which results in a high breadth ratio, low niche stability, and niche similarity. Both maize and its wild progenitors originated in Mexico; therefore, maize in Mexico experienced climatic conditions similar to those experienced by its wild progenitors. Additionally, maize has had fewer opportunities to adapt to diverse climatic conditions in Mexico compared with at a global scale and hence showed weaker niche shifts. Therefore, the effects of scale and area of origin require consideration when studying niche shifts.

Range size has generally been observed to reflect species’ resource use, which is positively associated with niche breadth (Gaston & Spicer, 2001; Botts et al., 2013; Yu et al., 2016; Vincent et al., 2020); however, contrasting results have also been obtained (Slatyer, Hirst & Sexton, 2013; Hirst et al., 2017; Cai et al., 2021). Few studies have examined the effects of niche shifts on range dynamics. Our results showed that the niche breadth of maize at the global scale has increased by ca. 421% and 253% relative to its wild progenitors (lowland and highland teosintes, respectively) and that its range size has increased by ca. 1,244% and 3,211%, respectively. This suggests that respectively small changes in niche breadth can result in large range expansions. Therefore, niche expansion may be a highly sensitive indicator of range expansion. Range expansion is generally used to measure changes in the distribution of a species under global change scenarios, especially for risk the assessment of invasive species (*e.g*., Bellard et al., 2013; Merow et al., 2017; Michalak et al., 2018; Di Febbraro et al., 2019). This finding suggests that niche expansion could be a more useful indicator of invasion risk than range shifts, given that the former may be more sensitive than the latter.

A review by Sanchez, Rasmussen & Porter (2014) indicated that the minimum and maximum temperatures for maize plants were 6.2 °C and 42.0 °C, respectively. We found that minimum temperature of the coldest month and maximum temperature of the warmest month were −17.99 °C and 43.70 °C, respectively, in the global potential range of maize. These observations indicate that maximum temperature for maize from the review was close to that inferred from the global potential range of maize; however, there was a large difference between the minimum temperature from the review and that inferred from the global potential range of maize. This might be explained by the fact that the minimum temperature inferred from the global potential range of maize exceeded the minimum temperature typically experienced by maize plants during production, whereas the minimum and maximum temperatures from the review and the maximum temperature inferred from the global potential range were within range of temperatures typically experienced by maize plants during production. Our theoretical and ecological inferences from the niche dynamics and SDMs might not be fully consistent with observations during maize production.

## Conclusions

To conclude, maize exhibited considerable niche lability, as it expanded its niche to regions with wider ranges of climatic conditions rather than remaining confined to the original climatic niche of its wild progenitors. These findings suggest that maize may be able to tolerate relatively novel climatic conditions and may therefore be less strongly affected by climate change. Moreover, these findings also suggest that cultivation-induced niche shifts may enable maize to become a global staple crop, feeding a rapidly expanding population and adapting to changing climatic conditions. Niche shifts promote range shifts, and small changes in niche breadth had large effects on the magnitude of range shifts. Additional analyses are needed to determine the limits of the novel conditions that maize can tolerate, especially relative to projected climate change.

## Additional Information and Declarations

### Data Availability

The following information was supplied regarding data availability: The code and data used for all statistical analyses are available at Figshare: Yang, Rujing; Chao, Runyao; Gong, Xiang; Feng, Jianmeng (2022): Niche and range shifts between maize and its wild progenitor. figshare. Journal contribution. https://doi.org/10.6084/m9.figshare.18133661.v2.
